# Supplementary figures and images for: Catching SARS-CoV-2 by Sequence Hybridization: a Comparative Analysis
Source: mSystems. 2021 Aug 3;6(4):e00392-21. doi: 10.1128/mSystems.00392-21 (PMC8407296; doi:10.1128/mSystems.00392-21)

**a**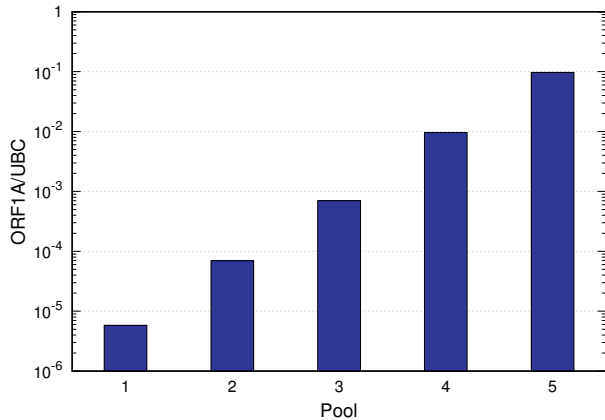**b**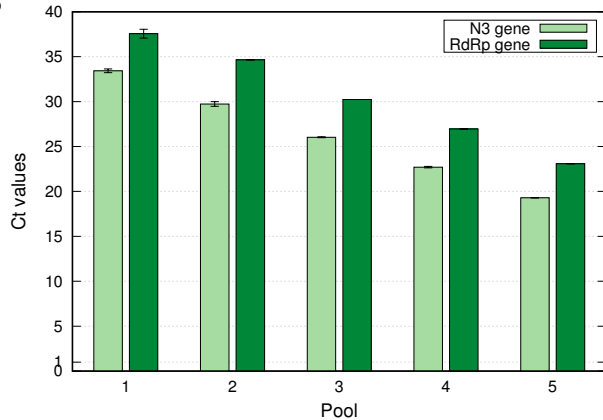

Supplement: FIG S1 [file msystems.00392-21-sf001.pdf]

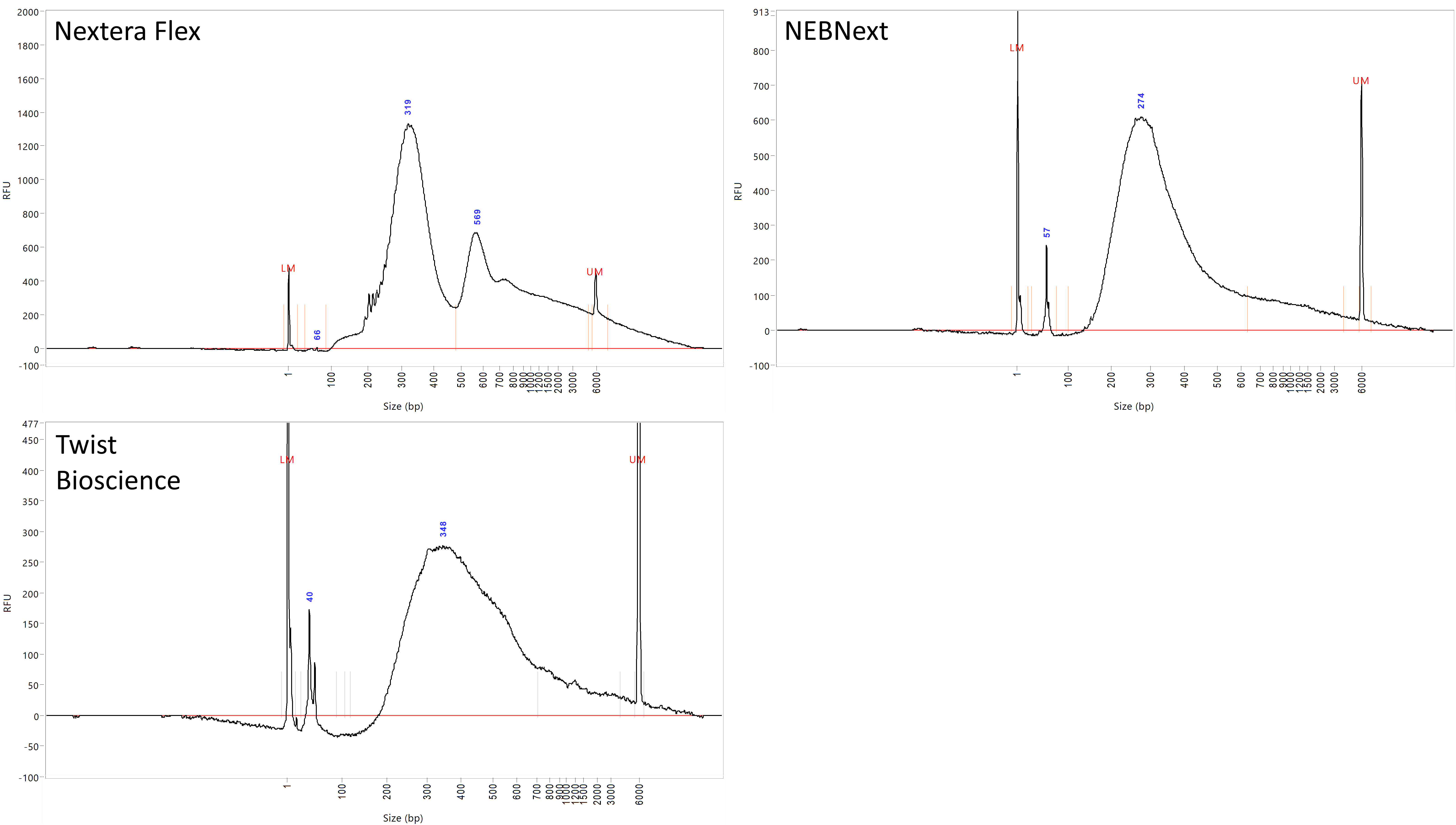

Supplement: FIG S2 [file msystems.00392-21-sf002.png]

**a**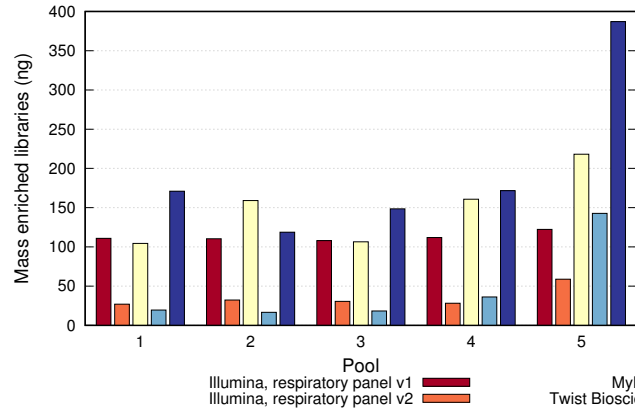**b**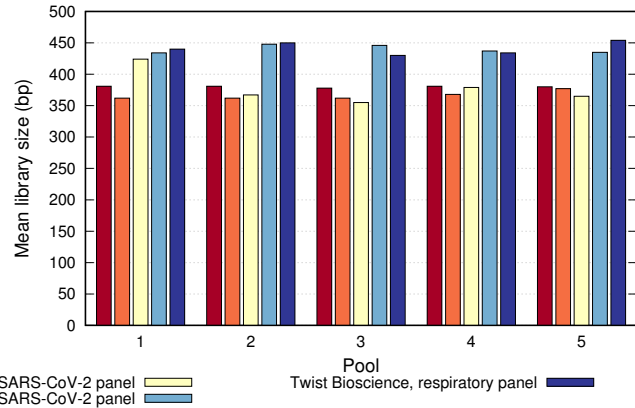

Supplement: FIG S3 [file msystems.00392-21-sf003.pdf]

Median coverage depth

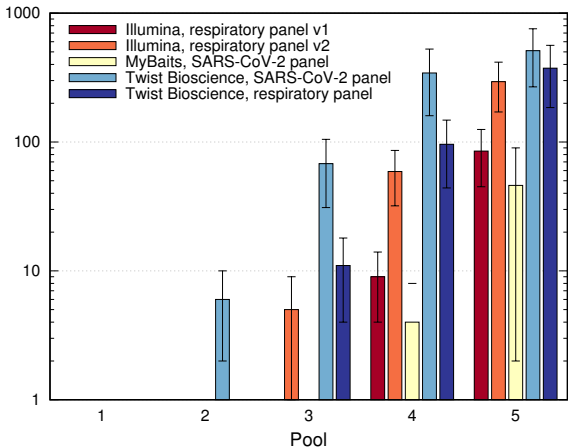

Supplement: FIG S4 [file msystems.00392-21-sf004.pdf]
