# Supplementary material for: Catching SARS-CoV-2 by Sequence Hybridization: a Comparative Analysis
Source: mSystems. 2021 Aug 3;6(4):e00392-21. doi: 10.1128/mSystems.00392-21 (PMC8407296; doi:10.1128/mSystems.00392-21)
Supplement: TABLE S2 [file msystems.00392-21-st002.docx]

| **Accession** | **Type** | **Gene** | **Product** | **Reads** |
| --- | --- | --- | --- | --- |
| NR_002728.3 | lncRNA | KCNQ1OT1 | KCNQ1 opposite strand/antisense transcript 1 | 825,039 |
| XM_024453098.1 | mRNA | TTN | titin | 301,068 |
| NR_146117.1 | rRNA | RNA45SN4 | RNA, 45S pre-ribosomal N4 | 227,341 |
| XM_005253351.3 | mRNA | GRIN2B | glutamate ionotropic receptor NMDA type subunit 2B | 222,456 |
| NR_003255.2 | asRNA | TSIX | TSIX transcript, XIST antisense RNA | 222,162 |
| NR_131012.1 | lncRNA | NEAT1 | nuclear paraspeckle assembly transcript 1 | 181,944 |
| NM_001135937.3 | mRNA | SMAD2 | SMAD family member 2 | 171,550 |
| NM_001347920.2 | mRNA | SNX19 | sorting nexin 19 | 154,836 |
| NM_173600.2 | mRNA | MUC19 | mucin 19, oligomeric | 151,992 |
| XM_017018270.1 | mRNA | AHNAK | AHNAK nucleoprotein | 148,448 |
| XM_024453100.1 | mRNA | TTN | titin | 143,548 |
| XR_002956694.1 | ncRNA | LOC112268022 | uncharacterized LOC112268022 | 140,790 |
| XR_001742414.1 | ncRNA | LOC107986350 | uncharacterized LOC107986350 | 139,164 |
| XR_001748874.1 | misc_RNA | KMT2D | lysine methyltransferase 2D | 137,151 |
| NM_001322468.1 | mRNA | MUC4 | mucin 4, cell surface associated | 136,944 |
| XM_017005895.2 | mRNA | NLGN1 | neuroligin 1 | 136,213 |
| XM_011514639.2 | mRNA | PTCHD4 | patched domain containing 4 | 131,334 |
| XM_011521106.1 | mRNA | CCDC168 | coiled-coil domain containing 168 | 128,058 |
| NM_001199319.2 | mRNA | PEX26 | peroxisomal biogenesis factor 26 | 127,617 |
| XM_017027494.1 | mRNA | MUC16 | mucin 16, cell surface associated | 127,296 |
| XM_017013716.1 | mRNA | ZFAT | zinc finger and AT-hook domain containing | 127,288 |
| XM_017009201.2 | mRNA | EBF1 | EBF transcription factor 1 | 125,130 |
| XM_017015929.1 | mRNA | ANKRD26 | ankyrin repeat domain 26 | 124,975 |
| NM_001376.5 | mRNA | DYNC1H1 | dynein cytoplasmic 1 heavy chain 1 | 119,640 |
| NM_001010854.2 | mRNA | TTC7B | tetratricopeptide repeat domain 7B | 116,826 |
| NM_004852.3 | mRNA | ONECUT2 | one cut homeobox 2 | 115,031 |
| NM_001346440.2 | mRNA | ERCC6 | ERCC excision repair 6, chromatin remodeling factor | 114,344 |
| NM_001164462.1 | mRNA | MUC12 | mucin 12, cell surface associated | 114,247 |
| NM_022055.2 | mRNA | KCNK12 | potassium two pore domain channel subfamily K member 12 | 108,512 |
| NR_146144.1 | rRNA | RNA45SN2 | RNA, 45S pre-ribosomal N2 | 106,520 |
