# Supplementary material for: Catching SARS-CoV-2 by Sequence Hybridization: a Comparative Analysis
Source: mSystems. 2021 Aug 3;6(4):e00392-21. doi: 10.1128/mSystems.00392-21 (PMC8407296; doi:10.1128/mSystems.00392-21)
Supplement: TABLE S3 [file msystems.00392-21-st003.docx]

| **Accession** | **Type** | **Gene** | **Product** | **Reads** |
| --- | --- | --- | --- | --- |
| NR_002728.3 | lncRNA | KCNQ1OT1 | KCNQ1 opposite strand/antisense transcript 1 | 825,039 |
| XM_024453100.1 | mRNA | TTN | titin | 502,418 |
| NM_001322468.1 | mRNA | MUC4 | mucin 4, cell surface associated | 502,128 |
| NM_003319.4 | mRNA | TTN | titin | 492,174 |
| XM_024453098.1 | mRNA | TTN | titin | 401,424 |
| NM_002458.3 | mRNA | MUC5B | mucin 5B, oligomeric mucus/gel-forming | 358,220 |
| NM_173600.2 | mRNA | MUC19 | mucin 19, oligomeric | 354,648 |
| NM_001164462.1 | mRNA | MUC12 | mucin 12, cell surface associated | 342,741 |
| NM_001278267.1 | mRNA | NBPF20 | NBPF member 20 | 332,100 |
| NM_001350929.2 | mRNA | AHNAK2 | AHNAK nucleoprotein 2 | 327,510 |
| XM_017017186.1 | mRNA | FAT3 | FAT atypical cadherin 3 | 263,136 |
| XR_001748874.1 | misc_RNA | KMT2D | lysine methyltransferase 2D | 254,709 |
| NM_001351365.1 | mRNA | NBPF19 | NBPF member 19 | 237,420 |
| XM_017027500.1 | mRNA | MUC16 | mucin 16, cell surface associated | 235,242 |
| NM_003890.2 | mRNA | FCGBP | Fc fragment of IgG binding protein | 229,698 |
| NM_001135937.3 | mRNA | SMAD2 | SMAD family member 2 | 205,860 |
| XM_017018270.1 | mRNA | AHNAK | AHNAK nucleoprotein | 204,116 |
| NM_001346440.2 | mRNA | ERCC6 | ERCC excision repair 6, chromatin remodeling factor | 200,102 |
| NM_001347920.2 | mRNA | SNX19 | sorting nexin 19 | 193,545 |
| NM_002016.2 | mRNA | FLG | filaggrin | 191,895 |
| NR_003255.2 | asRNA | TSIX | TSIX transcript, XIST antisense RNA | 185,135 |
| NM_001039703.6 | mRNA | NBPF10 | NBPF member 10 | 175,490 |
| NM_001304359.2 | mRNA | MUC5AC | mucin 5AC, oligomeric mucus/gel-forming | 174,480 |
| NR_146117.1 | rRNA | RNA45SN4 | RNA, 45S pre-ribosomal N4 | 173,849 |
| NR_146144.1 | rRNA | RNA45SN2 | RNA, 45S pre-ribosomal N2 | 173,095 |
| NM_015383.2 | mRNA | NBPF14 | NBPF member 14 | 171,360 |
| NR_133665.2 | ncRNA | MUC17 | mucin 17, cell surface associated | 170,400 |
| XM_017027495.1 | mRNA | MUC16 | mucin 16, cell surface associated | 169,728 |
| XR_002956694.1 | ncRNA | LOC112268022 | uncharacterized LOC112268022 | 168,948 |
| NM_003062.4 | mRNA | SLIT3 | slit guidance ligand 3 | 165,172 |
