# Supplementary material for: Catching SARS-CoV-2 by Sequence Hybridization: a Comparative Analysis
Source: mSystems. 2021 Aug 3;6(4):e00392-21. doi: 10.1128/mSystems.00392-21 (PMC8407296; doi:10.1128/mSystems.00392-21)
Supplement: TABLE S4 [file msystems.00392-21-st004.docx]

| **Accession** | **Type** | **Gene** | **Product** | **Reads** |
| --- | --- | --- | --- | --- |
| NR_002728.3 | lncRNA | KCNQ1OT1 | KCNQ1 opposite strand/antisense transcript 1 | 4,583,550 |
| XM_024453098.1 | mRNA | TTN | titin | 1,806,408 |
| NM_001347920.2 | mRNA | SNX19 | sorting nexin 19 | 1,316,106 |
| NM_001135937.3 | mRNA | SMAD2 | SMAD family member 2 | 1,269,470 |
| XM_024453100.1 | mRNA | TTN | titin | 1,148,384 |
| NR_003255.2 | asRNA | TSIX | TSIX transcript, XIST antisense RNA | 1,147,837 |
| NM_001354997.3 | mRNA | SLC35E3 | solute carrier family 35 member E3 | 1,127,104 |
| XM_005253351.3 | mRNA | GRIN2B | glutamate ionotropic receptor NMDA type subunit 2B | 1,084,473 |
| XR_002956694.1 | ncRNA | LOC112268022 | uncharacterized LOC112268022 | 1,041,846 |
| NM_001346146.2 | mRNA | FLRT2 | fibronectin leucine rich transmembrane protein 2 | 969,354 |
| NM_152318.3 | mRNA | C12orf45 | chromosome 12 open reading frame 45 | 964,484 |
| NR_131012.1 | lncRNA | NEAT1 | nuclear paraspeckle assembly transcript 1 | 886,977 |
| NM_001346440.2 | mRNA | ERCC6 | ERCC excision repair 6, chromatin remodeling factor | 886,166 |
| NM_024577.4 | mRNA | SH3TC2 | SH3 domain and tetratricopeptide repeats 2 | 846,976 |
| NM_001322238.2 | mRNA | RPS6KA5 | ribosomal protein S6 kinase A5 | 816,385 |
| XR_001742414.1 | ncRNA | LOC107986350 | uncharacterized LOC107986350 | 811,790 |
| XM_011521106.1 | mRNA | CCDC168 | coiled-coil domain containing 168 | 789,691 |
| NM_052898.2 | mRNA | XKR4 | XK related 4 | 789,399 |
| NM_173664.6 | mRNA | ARL10 | ADP ribosylation factor like GTPase 10 | 779,472 |
| XM_017003372.2 | mRNA | CPO | carboxypeptidase O | 775,797 |
| NM_024594.4 | mRNA | PANK3 | pantothenate kinase 3 | 770,550 |
| NM_001354704.2 | mRNA | TF | transferrin | 759,734 |
| NM_001351287.2 | mRNA | MGAT4C | MGAT4 family member C | 756,540 |
| XM_017014002.1 | mRNA | CYP7B1 | cytochrome P450 family 7 subfamily B member 1 | 751,506 |
| NM_032522.5 | mRNA | ZBTB37 | zinc finger and BTB domain containing 37 | 750,048 |
| NM_001164462.1 | mRNA | MUC12 | mucin 12, cell surface associated | 718,124 |
| NM_001304990.1 | mRNA | SPRY3 | sprouty RTK signaling antagonist 3 | 708,077 |
| NM_002458.3 | mRNA | MUC5B | mucin 5B, oligomeric mucus/gel-forming | 698,529 |
| NM_001010854.2 | mRNA | TTC7B | tetratricopeptide repeat domain 7B | 681,485 |
| XR_001747646.1 | ncRNA | LINC02666 | long intergenic non-protein coding RNA 2666 | 676,420 |
