# Supplementary material for: Catching SARS-CoV-2 by Sequence Hybridization: a Comparative Analysis
Source: mSystems. 2021 Aug 3;6(4):e00392-21. doi: 10.1128/mSystems.00392-21 (PMC8407296; doi:10.1128/mSystems.00392-21)
Supplement: TABLE S5 [file msystems.00392-21-st005.docx]

| **Pool** | **reads mapped** | **read mapping ratio** | **read mapping ratio stdev** | **# of bases uncovered** | **# of bases with min. coverage of 5x** | **# of bases with min. coverage of 10x** | **# of bases with min. coverage of 20x** | **median coverage** | **coverage stdev** | **breadth of coverage** | **breadth of coverage stdev** | **breadth of min. coverage of 20x** | **breadth of min. coverage of 20x stdev** |
| --- | --- | --- | --- | --- | --- | --- | --- | --- | --- | --- | --- | --- | --- |
| NF1.1 | 2 | 0 | 0 | 29,675 | 0 | 0 | 0 | 0 | 0.10 | 0.8% | 0.8% | 0% | 0% |
| NF1.2 | 15 | 0 | 0 | 28,543 | 0 | 0 | 0 | 0 | 0.32 | 4.5% | 1.5% | 0% | 0% |
| NF1.3 | 209 | 0.002 | 0 | 15,538 | 576 | 0 | 0 | 0 | 1.30 | 48.0% | 2.0% | 0% | 0% |
| NF1.4 | 2,161 | 0.017 | 0.001 | 182 | 25,551 | 14,033 | 2,106 | 9 | 5.86 | 99.4% | 0.1% | 7.0% | 1.9% |
| NF1.5 | 19,905 | 0.153 | 0.003 | 48 | 29,847 | 29,835 | 29,815 | 85 | 40.80 | 99.8% | 0% | 99.7% | 0.1% |
| NF2.1 | 9 | 0 | 0 | 29,087 | 0 | 0 | 0 | 0 | 0.25 | 2.7% | 0.4% | 0% | 0% |
| NF2.2 | 95 | 0.001 | 0 | 22,291 | 31 | 0 | 0 | 0 | 0.86 | 25.5% | 0.8% | 0% | 0% |
| NF2.3 | 1,329 | 0.010 | 0 | 842 | 18,065 | 5,069 | 185 | 5 | 3.95 | 97.2% | 0.8% | 0.6% | 0.4% |
| NF2.4 | 14,068 | 0.108 | 0.003 | 43 | 29,852 | 29,835 | 29,718 | 60 | 27.39 | 99.9% | 0% | 99.4% | 0.2% |
| NF2.5 | 69,446 | 0.534 | 0.011 | 20 | 29,866 | 29,851 | 29,847 | 295 | 123.79 | 99.9% | 0% | 99.8% | 0% |
| TB1.1 | 112 | 0.001 | 0 | 20,723 | 65 | 0 | 0 | 0 | 0.93 | 30.7% | 2.6% | 0% | 0% |
| TB1.2 | 1,360 | 0.010 | 0 | 1,307 | 18,217 | 5,829 | 452 | 6 | 4.47 | 95.6% | 1.3% | 1.5% | 0.2% |
| TB1.3 | 16,025 | 0.123 | 0.004 | 26 | 29,856 | 29,849 | 29,621 | 68 | 37.05 | 99.9% | 0% | 99.1% | 0.6% |
| TB1.4 | 81,290 | 0.625 | 0.003 | 2 | 29,881 | 29,867 | 29,858 | 343 | 182.97 | 100.0% | 0% | 99.8% | 0% |
| TB1.5 | 119,402 | 0.918 | 0.005 | 1 | 29,895 | 29,885 | 29,860 | 513 | 244.36 | 100.0% | 0% | 99.9% | 0% |
| TB2.1 | 22 | 0 | 0 | 27,629 | 0 | 0 | 0 | 0 | 0.39 | 7.6% | 2.0% | 0% | 0% |
| TB2.2 | 208 | 0.002 | 0 | 14,401 | 524 | 0 | 0 | 1 | 1.25 | 51.8% | 3.3% | 0% | 0% |
| TB2.3 | 2,557 | 0.020 | 0.002 | 171 | 26,806 | 17,761 | 3,640 | 11 | 7.04 | 99.4% | 0.4% | 12.2% | 2.7% |
| TB2.4 | 22,447 | 0.173 | 0.001 | 25 | 29,862 | 29,859 | 29,852 | 96 | 52.29 | 99.9% | 0% | 99.8% | 0% |
| TB2.5 | 86,543 | 0.666 | 0.007 | 2 | 29,884 | 29,869 | 29,860 | 374 | 189.75 | 100.0% | 0% | 99.9% | 0% |
| MB1.1 | 4 | 0 | 0 | 29,650 | 0 | 0 | 0 | 0 | 0.11 | 0.8% | 0.7% | 0% | 0% |
| MB1.2 | 10 | 0 | 0 | 29,209 | 0 | 0 | 0 | 0 | 0.24 | 2.3% | 0.9% | 0% | 0% |
| MB1.3 | 207 | 0.002 | 0 | 18,494 | 783 | 0 | 0 | 0 | 1.38 | 38.2% | 6.4% | 0% | 0% |
| MB1.4 | 1,236 | 0.010 | 0.001 | 2,439 | 14,346 | 4,018 | 675 | 4 | 4.84 | 91.8% | 4.2% | 2.3% | 1.1% |
| MB1.5 | 13,297 | 0.102 | 0.011 | 15 | 29,831 | 29,712 | 28,961 | 47 | 44.27 | 99.9% | 0% | 96.9% | 1.2% |
