# Supplementary material for: Catching SARS-CoV-2 by Sequence Hybridization: a Comparative Analysis
Source: mSystems. 2021 Aug 3;6(4):e00392-21. doi: 10.1128/mSystems.00392-21 (PMC8407296; doi:10.1128/mSystems.00392-21)
Supplement: TABLE S6 [file msystems.00392-21-st006.docx]

| **Pools** | **reads** | **# of mapped reads** | **mapping ratio** | **mapping ratio stdev** | **# of uncovered bases** | **median coverage** | **coverage stdev** | **# of duplicates** | **duplication ratio** | **duplication ratio stdev** | **median insert size** | **insert size absdev** |
| --- | --- | --- | --- | --- | --- | --- | --- | --- | --- | --- | --- | --- |
| NF1.1 | 387,201 | 6 | 0 | 0 | 29,356 | 0 | 0.20 | 55,483 | 13.7% | 2.5% | 179 | 38 |
| NF1.2 | 404,665 | 47 | 0 | 0 | 25,655 | 0 | 0.58 | 60,613 | 14.3% | 2.7% | 195 | 57 |
| NF1.3 | 318,938 | 434 | 0.001 | 0 | 8,273 | 2 | 1.92 | 42,861 | 12.9% | 2.2% | 190 | 55 |
| NF1.4 | 368,631 | 5,201 | 0.014 | 0 | 64 | 22 | 12.20 | 55,707 | 13.1% | 4.2% | 190 | 55 |
| NF1.5 | 425,407 | 58,886 | 0.138 | 0.002 | 29 | 252 | 119.12 | 51,545 | 12.1% | 0.1% | 186 | 53 |
| NF2.1 | 243,601 | 19 | 0 | 0 | 28,024 | 0 | 0.36 | 25,274 | 10.1% | 1.4% | 198 | 53 |
| NF2.2 | 310,508 | 215 | 0.001 | 0 | 16,553 | 0 | 1.46 | 39,885 | 12.6% | 1.4% | 187 | 56 |
| NF2.3 | 358,206 | 3,394 | 0.009 | 0 | 132 | 14 | 7.96 | 51,102 | 13.6% | 2.3% | 179 | 54 |
| NF2.4 | 450,137 | 45,798 | 0.102 | 0.004 | 7 | 191 | 90.14 | 60,075 | 13.3% | 0.7% | 179 | 52 |
| NF2.5 | 398,221 | 211,794 | 0.533 | 0.011 | 4 | 881 | 407.06 | 36,769 | 9.0% | 1.2% | 175 | 52 |
| TB1.1 | 599,686 | 796 | 0.001 | 0 | 10,735 | 2 | 4.85 | 115,858 | 19.3% | 0.1% | 198 | 66 |
| TB1.2 | 403,749 | 5,396 | 0.013 | 0 | 118 | 22 | 16.51 | 67,141 | 16.6% | 0.9% | 196 | 53 |
| TB1.3 | 473,105 | 74,300 | 0.157 | 0.003 | 3 | 314 | 178.63 | 93,305 | 19.7% | 0.9% | 220 | 53 |
| TB1.4 | 532,279 | 352,940 | 0.663 | 0.004 | 2 | 1,489 | 835.85 | 97,312 | 18.2% | 1.3% | 225 | 49 |
| TB1.5 | 514,459 | 473,865 | 0.920 | 0.005 | 0 | 2,028 | 991.91 | 19,786 | 3.8% | 0.4% | 234 | 53 |
| TB2.1 | 550,977 | 108 | 0 | 0 | 21,216 | 0 | 0.99 | 27,749 | 5.0% | 0.5% | 246 | 56 |
| TB2.2 | 513,539 | 825 | 0.002 | 0 | 3,531 | 3 | 3.30 | 23,719 | 4.6% | 0.3% | 247 | 60 |
| TB2.3 | 534,766 | 10,163 | 0.019 | 0.002 | 26 | 43 | 25.45 | 28,946 | 5.3% | 0.5% | 236 | 52 |
| TB2.4 | 505,207 | 89,001 | 0.176 | 0.001 | 5 | 383 | 211.93 | 23,548 | 4.6% | 0.3% | 238 | 53 |
| TB2.5 | 512,612 | 342,584 | 0.668 | 0.009 | 0 | 1,476 | 764.89 | 13,475 | 2.6% | 0.2% | 245 | 57 |
| MB1.1 | 979,277 | 32 | 0 | 0 | 27,397 | 0 | 0.37 | 81,044 | 8.2% | 0.6% | 171 | 62 |
| MB1.2 | 944,315 | 72 | 0 | 0 | 24,647 | 0 | 0.66 | 50,917 | 5.2% | 0.7% | 163 | 58 |
| MB1.3 | 772,647 | 1,179 | 0.002 | 0 | 2,715 | 4 | 4.06 | 35,239 | 4.6% | 0.2% | 144 | 47 |
| MB1.4 | 1,071,878 | 10,447 | 0.010 | 0.001 | 18 | 37 | 32.47 | 69,116 | 6.4% | 0.7% | 158 | 46 |
| MB1.5 | 1,049,216 | 103,973 | 0.099 | 0.011 | 2 | 369 | 341.53 | 63,835 | 6.1% | 0.5% | 165 | 47 |
